# Chemoimmunotherapy Combinations in Elderly Patients with Metastatic Non-Small Cell Lung Cancer and PD-L1 Expression < 50%: Results from an Italian Real-World Study

**DOI:** 10.3390/jcm15031165

**Published:** 2026-02-02

**Authors:** Antonello Veccia, Ettore D’Argento, Floriana Morgillo, Elio Gregory Pizzutilo, Fabiana Vitiello, Alberto Pavan, Fiorella Lombardo, Marco Russano, Alessandro Morabito, Eleonora Gariazzo, Carlo Genova, Rita Chiari, Antonella Cristofano, Alessandro Delconte, Emanuela Vattemi, Alessandra Dessi, Daniele Galanti, Simona Busato, Giovanni Palazzolo, Clementina Savastano, Giuseppe Azzarello, Francesco Verderame, Cristina Mazzi, Daniela Bencardino, Mariachiara Dipasquale, Alessandro Scala, Carminia Maria Della Corte, Daniele Piscazzi, Marina Gilli, Emilio Bria, Orazio Caffo, Stefania Gori, Alessandro Inno

**Affiliations:** 1 Medical Oncology, Santa Chiara Hospital, 38122 Trento, Italy; 2 Comprehensive Cancer Center, Fondazione Policlinico Universitario Agostino Gemelli IRCCS, 00168 Rome, Italy; 3 Department of Precision Medicine, Università Degli Studi Della Campania “Luigi Vanvitelli”, 80131 Naples, Italy; 4 S.C. Oncologia Falck, Dipartimento Ematologia, Oncologia e Medicina Molecolare, Niguarda Cancer Center, Grande Ospedale Metropolitano Niguarda, 20162 Milano, Italy; 5 Pulmonary Oncology Unit, Department of Pneumology and Oncology, A.O. Dei Colli Monaldi Hospital, 80131 Naples, Italy; 6 Medical Oncology Department, AULSS 3 Serenissima, Dell’Angelo General Hospital, Mestre and SS Giovanni e Paolo General Hospital, 30174 Venice, Italy; 7 Lung Unit, Ospedale Pederzoli, Peschiera del Garda, 37019 Verona, Italy; 8 Medical Oncology, Fondazione Policlinico Universitario Campus Bio-Medico, 00128 Roma, Italy; 9 Thoracic Medical Oncology, Istituto Nazionale Tumori—IRCCS-Fondazione G. Pascale, 80131 Naples, Italy; 10 Medical Oncology, Santa Maria della Misericordia Hospital, University of Perugia, 06123 Perugia, Italy; 11 Department of Internal Medicine and Medical Specialties, University of Genoa, 16126 Genoa, Italy; 12 Academic Medical Oncology Unit, IRCCS Ospedale Policlinico San Martino, 16132 Genoa, Italy; 13 Medical Oncology Unit, AST1, 61121 Pesaro, Italy; 14 Oncology and Onco-Hematology Department, Ospedale Generale Regionale F. Miulli, Acquaviva Delle Fonti, 70021 Bari, Italy; 15 Department of Medical Oncology, CRO di Aviano, National Cancer Institute, IRCCS, 33081 Aviano, Italy; 16 Medical Oncology, Azienda Sanitaria Dell’alto Adige, 39100 Bolzano, Italy; 17 S.C. Oncologia Medica, Ospedale Armando Businco ARNAS Brotzu, 09121 Cagliari, Italy; 18 Medical Oncology Unit, Ospedale Buccheri La Ferla Fatebenefratelli, 90123 Palermo, Italy; 19 Medical Oncology and Hematology, AULSS 3 Serenissima, 30015 Venice, Italy; 20 Division of Medical Oncology, AULSS 6 Cittadella, 35013 Cittadella, Italy; 21 Medical Oncology Unit, San Giovanni di Dio e Ruggi D’aragona, 84131 Salerno, Italy; 22 Medical Oncology Unit, AULSS 3 Serenissima, 30174 Venice, Italy; 23 Oncology Unit, AO Ospedali Riuniti “Villa Sofia-Cervello”, 90146 Palermo, Italy; 24 Clinical Research Unit, IRCCS Sacro Cuore Don Calabria Hospital, 37024 Negrar di Valpolicella, Italy; 25 Scientific Direction, IRCCS Sacro Cuore Don Calabria Hospital, 37024 Negrar di Valpolicella, Italy; 26 Ospedale Isola Tiberina-Gemelli Isola, Università Cattolica del Sacro Cuore, 00168 Roma, Italy; 27 Medical Oncology Unit, IRCCS Sacro Cuore Don Calabria Hospital, 37024 Negrar di Valpolicella, Italy

**Keywords:** elderly, chemotherapy, immunotherapy, NSCLC, PD-L1, survival, safety

## Abstract

**Background:** Chemoimmunotherapy combinations represent the standard first-line treatment for non-oncogene addicted metastatic NSCLC (mNSCLC). However, evidence in elderly patients remains limited and conflicting. We conducted an analysis of the efficacy and safety of chemoimmunotherapy in patients aged ≥75 years enrolled in the Real-Combo Lung study, an observational study including patients with non-oncogene-addicted mNSCLC and PD-L1 expression < 50%. **Patients and Methods:** The primary objective of the study was to compare progression-free survival (PFS) and overall survival (OS) between patients aged ≥75 (elderly cohort) and those aged <75 years (non-elderly cohort). Safety outcomes were evaluated as a secondary objective. **Results:** A total of 495 patients were enrolled, with 89 (18%) aged ≥75 and 406 (82%) aged <75 years. No significant differences in PFS and OS were observed between the two cohorts. The median PFS was 13.3 months (95% CI: 9.3–NR) in the elderly cohort and 10.5 months (95% CI: 9.5–12.9) in the non-elderly cohort (unadjusted HR 0.84, 95% CI: 0.61–1.16, *p* = 0.29). The median OS was 17.5 months (95% CI: 14.7–NR) versus 21.4 months (95% CI: 17–NR), respectively (unadjusted HR 1.09, 95% CI: 0.76–1.56, *p* = 0.63). In multivariable analysis, ECOG PS ≥ 2 and baseline use of steroids were significantly associated with a worse outcome in the elderly cohort for both PFS and OS. Safety data did not differ significantly between cohorts. **Conclusions:** In this real-world study, elderly patients with mNSCLC derived outcomes comparable to those of younger patients, with similar efficacy and a manageable safety profile when treated with chemoimmunotherapy combinations.

## 1. Introduction

Lung cancer remains one of the most frequently diagnosed malignant tumours worldwide and the leading cause of cancer deaths [1]. Approximately 70% of cases are classified as non-small cell lung cancer (NSCLC), most of which are diagnosed at an advanced stage, with a median age at diagnosis of 71 years [2]. Excluding oncogene-addicted tumours that receive targeted therapy as first-line treatment, patients with non-oncogene-addicted NSCLC are candidates for a chemoimmunotherapy or single-agent immunotherapy when programmed death ligand-1 (PD-L1) expression levels are < 50% and ≥50%, respectively [3,4]. The most commonly used chemo-immunotherapy regimens that have demonstrated an overall survival (OS) benefit in randomized phase 3 studies include: (i) the anti-PD-1 agents (pembrolizumab or cemiplimab) combined with histology-driven platinum-based chemotherapy (4 cycles), followed by maintenance with the anti-PD-1 agent (in combination with pemetrexed in non-squamous histology) [5,6,7]; and (ii) dual immune checkpoint blockade with anti-PD-1 and anti-CTLA-4 antibodies (nivolumab plus ipilimumab), combined with a short course of platinum-based chemotherapy (2 cycles), followed by maintenance with nivolumab plus ipilimumab [8].

However, patients enrolled in these pivotal trials had a good performance status (ECOG PS 0–1) and a median age of approximately 60 years, which differs substantially from the demographic profile of patients treated in real-world practice. Patients aged ≥75 years accounted for less than 10% of the study populations, limiting the strength of evidence supporting the use of chemoimmunotherapy in this subgroup [9,10]. Specifically, Keynote 189 and EMPOWER-Lung3 reported statistically significant OS benefits with chemoimmunotherapy in older patients, but the age cut-off was 65 years. In contrast, in the subgroup analysis of Keynote 407, patients ≥ 65 years did not show a statistically significant benefit from chemoimmunotherapy (HR 0.74; 95% CI 0.51–1.62), although the HR was numerically favourable to the experimental arm [6]. Similarly, in the CheckMate 9LA study, which included 70 patients (10%) aged ≥75 years, the treatment effect did not reach statistical significance and the HR did not favour the experimental arm receiving dual ICI blockade plus chemotherapy (HR 1.21, 95%CI 0.69–2.12) [8].

Conflicting evidence has also emerged from retrospective real-world studies comparing the efficacy and safety of chemoimmunotherapy between the elderly (≥75 years) and non-elderly population with mNSCLC. One study reported comparable survival outcomes and rates of immune-related adverse events (irAEs) [11], while two studies found similar survival but a higher incidence of adverse events among the elderly [12,13]. Conversely, in the study of Morimoto et al., the pemetrexed-based regimen, but not the paclitaxel-based regimen was associated with poorer outcomes in the elderly patients [14].

Overall, these findings suggest that age-related immunosenescence alone may not explain the reduced activity of immunotherapy-based combinations in older patients. Chronological and biological age should be clearly distinguished, and, as recommended by the International Society of Geriatric Oncology (SIOG), a comprehensive geriatric assessment (CGA) should be performed before proposing systemic treatment to patients aged ≥70 years [15].

Therefore, evaluating the outcomes of chemoimmunotherapy in elderly patients represents an unmet need. Here we report an analysis on efficacy and safety of chemoimmunotherapy in elderly patients included in the Real-Combo Lung study [16], an observational study including patients with non-oncogene-addicted metastatic NSCLC and PD-L1 expression < 50%.

## 2. Methods

The Real-Combo Lung study is an observational, ambispective, multicentre study conducted across 23 Italian oncology centres from 1 April 2022 to 31 December 2023. It included metastatic NSCLC patients who were wild type for *EGFR* and *ALK* alterations and had a PD-L1 ≤ 50%. The patients received combinations of chemotherapy and immune checkpoint inhibitors that were approved by the Italian National Health System within that timeframe: pembrolizumab plus platinum-based chemotherapy and nivolumab/ipilimumab plus platinum-based chemotherapy. Patients were divided into two cohorts according to age: the elderly cohort (≥75 years) and the non-elderly cohort (<75 years). This analysis is based on data updated as of 30 June 2024. Clinical data were collected anonymously through a dedicated clinical case report form specifically designed for the study.

The primary objective of this analysis was to compare the efficacy of chemoimmunotherapy combinations in terms of PFS and OS between elderly and non-elderly patients. PFS and OS were defined as the time elapsed from the start of treatment to disease progression and death from any cause, respectively. The date of their last follow-up was used to censor patients who did not develop events. Safety assessment was one of the secondary objectives of the study: the treatment-related adverse events were recorded according to version 5.0 of the National Cancer Institute Common Terminology Criteria for Adverse Events (NCI CTCAE). Exploratory analyses concerned the difference between the clinical characteristics and outcomes of elderly patients compared to non-elderly patients receiving various combinations of chemotherapy plus immune checkpoint inhibitors; moreover, the assessment of potential prognostic factors within the elderly population.

Pearson’s Chi-squared test or Fisher’s exact test were used to compare categorical variables that were shown by frequency and percentage. Age was shown in median and range, while the Wilcoxon rank sum test compared the two age groups. PFS and OS were estimated using the Kaplan–Meier method, and median values with 95% confidence intervals (CIs) were calculated according to age and treatment cohort. Survival curves were compared using the log-rank test. Risk factors for PFS and OS—including type of combination treatment, PD-L1 expression, ECOG status, histology, baseline steroid use and number of metastatic sites—were first evaluated by univariable Cox proportional hazards regression, followed multivariable analysis including variables with *p*-values < 0.1. The proportional hazards assumption was verified using weighted Schoenfeld residuals. Two-sided *p*-values < 0.05 were considered statistically significant. All analyses were performed using R statistical software (version 4.4.1) [17].

The study protocol was approved by the ethics committee at each participating centre, and the study was conducted in accordance with Good Clinical Practice guidelines. Written informed consent was obtained from all patients.

## 3. Results

A total of 495 patients with mNSCLC who received first-line chemoimmunotherapy were included in the analysis. Of these, 89 (18%) were aged ≥75 years (elderly cohort) and 406 (82%) were <75 years (non-elderly cohort). The baseline characteristics of the study population are summarized in Table 1. The median age was 77 years (range 75, 89) in the elderly-cohort and 66 years (range 29, 74) in the non-elderly cohort. The two cohorts were comparable in terms of histological subtypes, ECOG PS, smoking history, number of comorbidities, PDL1 expression levels and number and sites of metastases, except for central nervous system metastases, which were significantly less frequent in the elderly cohort. Conversely, significant differences were observed in other baseline features: compared with patients < 75 years, those in the elderly cohort were more frequently male (76.4% vs. 62.3%, *p* = 0.012), more commonly treated with pembrolizumab plus chemotherapy (80.9% vs. 68%, *p* = 0.016) and less likely to receive concomitant steroid therapy (17.5% vs. 30.3%, *p* = 0.021). Steroids were generally used for untreated or symptomatic brain metastases, dyspnoea due to pleural effusion and as supportive therapy (pain and loss of appetite).

**Figure 1:**
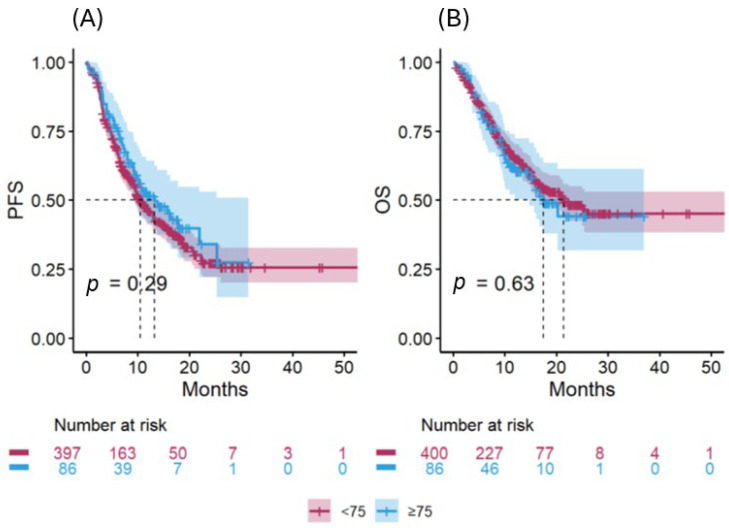
PFS (**A**) and OS (**B**) in the two treatment cohorts, according to the age.

No significant differences in PFS or OS were observed between the two age cohorts (Figure 1A,B). The median PFS was 13.3 months (95% CI: 9.3–NR) in the elderly cohort and 10.5 months (95% CI: 9.5–12.9) in the non-elderly cohort (unadjusted HR 0.84, 95% CI: 0.61–1.16, *p* = 0.29). The median OS was 17.5 months (95% CI: 14.7–NR) and 21.4 months (95% CI: 17–NR), respectively (unadjusted HR 1.09, 95% CI: 0.76–1.56, *p* = 0.63).

**Figure 2:**
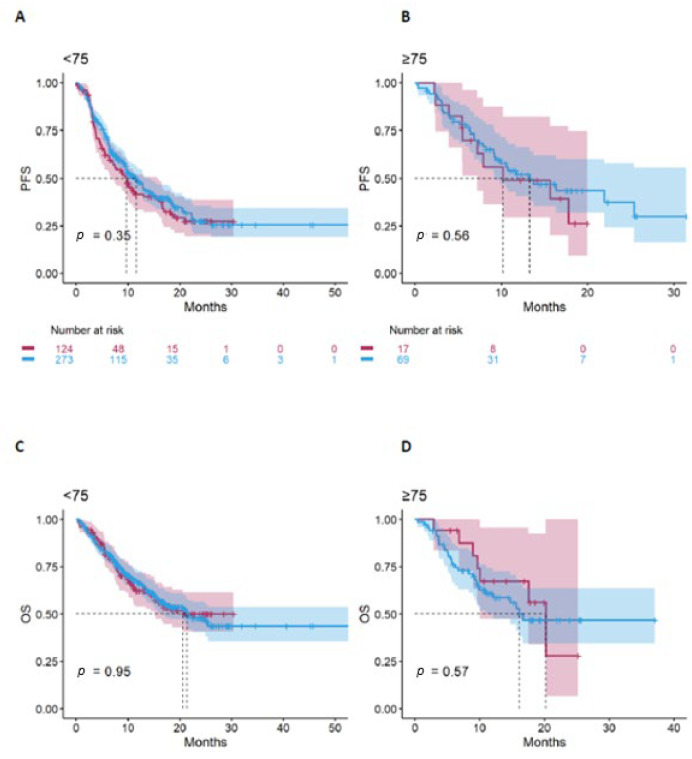
PFS in non-elderly (**A**) and elderly (**B**) cohorts, and OS in non-elderly (**C**) and elderly (**D**) cohorts according to the type of combination treatment.

In both cohorts, the type of chemoimmunotherapy treatment did not significantly affect PFS (Figure 2A,B) or OS (Figure 2C,D). Specifically, among elderly patients, there was no difference between those treated with pembrolizumab plus chemotherapy and those treated with ipilimumab/nivolumab plus chemotherapy in terms of PFS (*p* = 0.56) or OS (*p* = 0.57).

In the univariate analysis, no association of sex and/or brain metastases with survival outcomes (PFS and OS) was found in the elderly subgroup.

In the multivariate analysis of the elderly cohort, ECOG PS ≥ 2 and baseline corticosteroid use were independently associated with poorer outcomes for both PFS and OS. PDL1 expression levels of 1–49% were also associated with shorter PFS but not OS. Results of the multivariable analysis for PFS and OS are presented in Table 2 and Table 3, respectively.

With regards to safety, the incidence of chemotherapy-related adverse events of any grade was significantly higher in patients ≥ 75 years compared with younger patients (74.2% vs. 61.6%, *p* = 0.025), whereas grade 3–4 toxicity occurred at similar rates (14.6% vs. 14.5%, *p* > 0.9) (Table 4). The incidence of immune-related adverse events (irAEs) did not differ significantly between the elderly and non-elderly cohorts: any grade irAEs occurred in 42.7% and 41.6% of patients (*p* = 0.9), and grade 3–4 irAEs developed in 11.2% and 12.3% of patients, respectively (*p* = 0.8).

Treatment discontinuations due to adverse events were significantly less frequent among elderly patients: 6 (6.7%) versus 63 (15.5%) in the non-elderly cohort (*p* = 0.030). No treatment-related deaths occurred in the elderly cohort, whereas 2 (1.7%) were reported in the non-elderly cohort.

## 4. Discussion

This analysis of the Real-Combo Lung study [16] evaluated the efficacy and safety of first-line chemoimmunotherapy in patients with mNSCLC aged ≥75 years compared to younger patients. Among 495 patients, 89 (18%) were ≥75 years. Survival outcomes were similar between the two age cohorts, with no significant differences in PFS and OS, regardless of the chemoimmunotherapy regimen used.

Our results are consistent with the findings of pivotal phase 3 trials such as Keynote 189 and EMPOWER-Lung3 [5,7], which demonstrated a statistically significant OS benefit for chemoimmunotherapy also in older patients, although these studies adopted a lower age cut-off (≥65 years). Conversely, Keynote 407 did not show a statistically significant advantage for chemoimmunotherapy in this subgroup [6], whereas the Checkmate-9LA study reported a HR for OS of 1.21 (95% CI 0.69–2.12) in patients aged ≥75, suggesting a limited or even absent benefit from chemoimmunotherapy compared to chemotherapy alone [8].

Moreover, in this latter study, no differences were observed between subgroups defined by the presence or absence of brain metastases or by gender. Elderly patients derived a survival benefit from chemoimmunotherapy, regardless of the presence or absence of brain involvement and patient sex [8].

Data from real-world studies remain heterogeneous. A retrospective Japanese study including 299 patients with non-squamous mNSCLC found no significant differences in PFS between patients aged <75 and those aged ≥75 years: 8.5 (09% CI, 7–9.9) and 8.9 (95% CI, 6.7–10.5), respectively [12]. Another Japanese study of 111 patients with mNSCLC (41 aged ≥75 years and 70 aged <75 years) also showed comparable efficacy between the two age cohorts: PFS was 5.6 vs. 6.3 months (*p* = 0.98), response rate 36.6% vs. 44.9% (*p* = 0.51), and DCR 80.5% vs. 76.8% (*p* = 0.83), for elderly and non-elderly patients, respectively. However, data on the association of age, brain metastases, histology and stage with survival were not reported in the multivariate analysis [11].

The largest retrospective study on patients aged ≥75 years with mNSCLC included 1245 patients and found that patients treated with chemo-immunotherapy (n = 354) did not report a survival benefit compared to those treated with immunotherapy alone (n = 425) [13]. Similar results were reported by other observational studies [18,19]. However, in these studies the control arm did not consist of non-elderly patients but of elderly patients receiving immunotherapy alone. In the study of Blasi et al., squamous histology and the presence of brain metastases were associated with worse PFS and OS in the multivariate analysis [18], while in that of Zhang et al., histology, sex and stage were also not associated with PFS and OS in the multivariate analysis [19].

Data from randomized clinical trials suggest that chemoimmunotherapy combinations may have limited efficacy in elderly patients with squamous histology compared to younger patients [6,8]. Conversely, a real-world study showed that elderly patients receiving pembrolizumab plus paclitaxel-based chemotherapy regimens for squamous mNSCLC achieved PFS and OS comparable to those of non-elderly patients, whereas outcomes were significantly poorer among elderly patients treated with pembrolizumab plus pemetrexed-based chemotherapy regimens for non-squamous histology [14].

In our study, however, survival outcomes were similar across histological subtypes and treatment regimens.

The elderly population in our study differed from younger patients in several characteristics: they were more frequently male, had a lower incidence of brain metastases, and were less likely to receive steroids at baseline, features that may reflect patient selection and partly explain their comparable survival outcomes. At the multivariable analysis, in the elderly cohort ECOG PS ≥ 2 and baseline use of steroids were independently associated with a worse prognosis, consistent with previous observations in real-world studies [12,14].

From a safety perspective, elderly patients experienced a higher incidence of any-grade chemotherapy-related adverse events, whereas grade 3–4 toxicity and irAEs occurred at similar frequencies across age groups. Toxicity-related treatment discontinuation and deaths were rare among elderly patients, supporting the feasibility of chemoimmunotherapy in this setting. These findings are consistent with previous reports and reinforce that age alone should not preclude combination treatment in carefully selected patients [11].

Nevertheless, treatment decisions in older patients must take into account frailty, comorbidities, functional status, organ function, cognitive status, and social support. SIOG guidelines recommend using the G8 tool for patients ≥ 70 years [20,21,22] to identify those requiring a comprehensive geriatric assessment [15]. Although our study did not include a prospective geriatric evaluation, the characteristics of enrolled patients suggest a relatively fit and select elderly population.

To our knowledge, this is the first real-world study that included elderly patients treated with either pembrolizumab- or nivolumab/ipilimumab-based chemoimmunotherapy. Although limited to patients with PDL1 < 50%, a group generally associated with a poorer prognosis, elderly patients achieved outcomes comparable to those of younger individuals.

Our study has several limitations, including potential selection bias, lack of frailty assessment and immature OS data due to limited follow-up. Adverse events may also have been underreported, and post-progression treatments were not evaluated. Despite these limitations, our findings support the real-world feasibility of chemoimmunotherapy in selected patients aged ≥75 years with mNSCLC.

## 5. Conclusions

In summary, this multicentre observational study indicates that, in clinical practice, elderly patients (≥75 years) with mNSCLC and PD-L1 < 50% derive comparable efficacy outcomes from first-line chemoimmunotherapy to their younger counterparts. Despite a higher incidence of chemotherapy-related adverse events, toxicity was manageable and did not compromise treatment feasibility. These results underscore that age alone should not preclude the use of chemoimmunotherapy in appropriately selected patients.

## Figures and Tables

**Table 1:** Baseline patient’s characteristics.

Characteristic	Overall N = 495 ^1^	<75 Years N = 406 (82%) ^1^	≥75 Years N = 89 (18%) ^1^	*p* ^2^
Age				-
Median (range)	68.2 (29–89)	66 (29, 74)	77 (75, 89)	
Sex—no. (%)				0.012
Male	321 (64.8)	253 (62.3)	68 (76.4)	
Female	174 (35.2)	153 (37.7)	21 (23.6)	
Smoking status—no (%)				0.3
Current/Former	439 (90.3)	356 (89.7)	83 (93.3)	
Never	47 (9.7)	41 (10.3)	6 (6.7)	
Unknown	9	9	0	
ECOG PS—no. (%)				>0.9
0–1	445 (90.1%)	365 (90.1)	80 (89.9)	
≥2	49 (9.9%)	40 (9.9)	9 (10.1)	
Unknown	1	1	0	
Number of comorbidities				0.9
0–2	250 (56.7)	200 (56.5)	50 (57.5)	
>2	191 (43.3)	154 (43.5)	37 (42.5)	
Unknown	54	52	2	
Baseline steroids				0.021
Yes	128 (28.1)	114 (30.3)	14 (17.5)	
No	328 (71.9)	262 (69.7)	66 (82.5)	
Unknown	39	30	9	
Histology—no. (%)				0.057
Non-Squamous	389 (79.1)	326 (80.7)	63 (71.6)	
Squamous	103 (20.9)	78 (19.3)	25 (28.4)	
Unknown	3	2	1	
PD-L1—no (%)				0.056
<1%	219 (44.9)	188 (46.9)	31 (35.6)	
1–49%	269 (55.1)	213 (53.1)	56 (64.4)	
Unknown	7	5	2	
Number of metastatic sites—no (%)				0.8
≤2	315 (67)	259 (67.3)	56 (65.9)	
>2	155 (33)	126 (32.7)	29 (34.1)	
Unknown	25	21		
Bone metastases—no (%)				>0.9
Yes	163 (34.5)	134 (34.4)	29 (34.9)	
No	309 (65.5)	255 (65.6)	54 (65.1)	
Unknown	23	17	6	
Liver metastases—no (%)				0.7
Yes	40 (8.5)	32 (8.2)	8 (9.6)	
No	431 (91.5)	356 (91.8)	75 (90.4)	
Unknown	24	18	6	
CNS metastases—no (%)				0.017
Yes	89 (18.8)	81 (20.8)	8 (9.5)	
No	385 (81.2)	309 (79.2)	76 (90.5)	
Unknown	21	16	5	
Stage				0.7
IVA	74 (15)	62 (15.3)	12 (13.5)	
IVB	419 (85)	342 (84.7)	77 (86.5)	
Unknown	2	2	0	

^1^ n/N, %; ^2^ Pearson’s Chi-squared test.

**Table 2:** Multivariable analysis for PFS.

	<75			≥75		
Variable	HR	95% CI	*p*-Value	HR	95% CI	*p*-Value
Treatment						
Ipi + Nivo	-	-		-	-	
Pembrolizumab	1.29	0.90–1.84	0.2	1.41	0.59–3.38	0.4
ECOG PS						
0–1	-	-		-	-	
2–3	1.59	1.00–2.52	0.051	3.30	1.27–8.58	0.014
PDL1—n (%)						
<1	-	-		-	-	
1–49	0.71	0.51–0.98	0.039	0.55	0.26–1.19	0.13
Baseline steroids						
No	-	-		-	-	
Yes	1.94	1.38–2.73	<0.001	3.06	1.28–7.31	0.012
Histology						
Non-squamous	-	-		-	-	
Squamous	1.78	1.22–2.61	0.003	0.67	0.27–1.68	0.4
Number of metastatic sites						
≤2	-	-		-	-	
>2	1.49	1.07–2.07	0.018	0.88	0.41–1.88	0.7

HR: Hazard Ratio, CI: Confidence Interval.

**Table 3:** Multivariable analysis for OS.

	<75			≥75		
Variable	HR	95% CI	*p*-Value	HR	95% CI	*p*-Value
Treatment						
Ipi + Nivo	-	-		-	-	
Pembrolizumab	1.05	0.78–1.42	0.8	1.01	0.47–2.17	>0.9
ECOG PS						
0–1	-	-		-	-	
2–3	1.75	1.17–2.60	0.006	3.04	1.21–7.65	0.018
PDL1—n (%)						
<1	-	-		-	-	
1–49	0.90	0.69–1.18	0.4	0.42	0.21–0.83	0.013
Baseline steroids						
No	-	-		-	-	
Yes	1.63	1.22–2.19	0.001	2.37	1.04–5.39	0.040
Histology						
Non-squamous	-	-		-	-	
Squamous	1.91	1.39–2.62	<0.001	0.69	0.31–1.52	0.4
Number of metastatic sites						
≤2	-	-		-	-	
>2	1.67	1.26–2.21	<0.001	0.93	0.48–1.82	0.8

HR: Hazard Ratio, CI: Confidence Interval.

**Table 4:** Incidence of adverse events.

Adverse Events	Overall, n (%) N = 495	<75, n (%) N = 406 (82)	≥75, n (%) N = 89 (18)	*p*-Value ^1^
*Chemotherapy-related*				
Any grade	316 (63.8)	250 (61.6)	66 (74.2)	0.025
G3–G4	72 (14.5)	59 (14.5)	13 (14.6)	>0.9
*Neutropenia*				
Any grade	92 (18.6)	78 (19.2)	14 (15.7)	0.4
G3–G4	32 (6.5)	27 (6.7)	5 (5.6)	0.7
*Thrombocytopenia*				
Any grade	34 (6.9)	24 (5.9)	10 (11.2)	0.072
G3–G4	8 (1.6)	6 (1.5)	2 (2.2)	0.6
*Anemia*				
Any grade	176 (35.6)	140 (34.5)	36 (40.4)	0.3
G3–G4	23 (4.6)	20 (4.9)	3 (3.4)	0.8
*Nausea/Vomiting*				
Any grade	91 (18.4)	77 (19.0)	14 (15.7)	0.5
G3–G4	5 (1.0)	4 (1.0)	1 (1.1)	>0.9
*Peripheral neuropathy*				
Any grade	29 (5.9)	21 (5.2)	8 (9.0)	0.2
G3–G4	0 (0.0)	0 (0.0)	0 (0.0)	-
***Immune-related***				
Any grade	207 (41.8)	169 (41.6)	38 (42.7)	0.9
G3–G4	60 (12.1)	50 (12.3)	10 (11.2)	0.8
*Endocrine*				
Any grade	48 (9.7)	40 (9.9)	8 (9.0)	0.8
G3–G4	7 (1.4)	6 (1.5)	1 (1.1)	>0.9
*Rash*				
Any grade	57 (11.5)	44 (10.8)	13 (14.6)	0.3
G3–G4	9 (1.8)	8 (2.0)	1 (1.1)	>0.9
*Hepatitis*				
Any grade	27 (5.5)	24 (5.9)	3 (3.4)	0.5
G3–G4	9 (1.8)	7 (1.7)	2 (2.2)	0.7
*Colitis*				
Any grade	56 (11.3)	44 (10.8)	12 (13.5)	0.4
G3–G4	16 (3.2)	13 (3.2)	3 (3.4)	>0.9
*Pneumonitis*				
Any grade	25 (5.1)	21 (5.2)	4 (4.5)	>0.9
G3–G4	8 (1.6)	6 (1.5)	2 (2.2)	0.6
*Nephritis*				
Any grade	19 (3.8)	16 (3.9)	3 (3.4)	>0.9
G3–G4	5 (1.0)	5 (1.2)	0 (0.0)	0.6
**Leading to discontinuation**	69 (13.9)	63 (15.5)	6 (6.7)	0.030
**Discontinuation of chemotherapy**	45 (67.2)	39 (63.9)	6 (100)	0.2
**Discontinuation of immunotherapy**	62 (89.9)	56 (88.9)	6 (100)	>0.9
**irAEs leading to death**	2 (1.4)	2 (1.7)	0 (0.0)	>0.9

^1^ Pearson’s Chi-squared test; Fisher’s exact test.

## Data Availability

All data supporting the results of the study can be found in the article. Researchers can contact the corresponding author of this article by email and indicate the required research materials and purpose. We will be glad to provide relevant materials for this study after approval and discussion.
